# Sex Differences in the Physiological and Behavioral Effects of Chronic Oral Methylphenidate Treatment in Rats

**DOI:** 10.3389/fnbeh.2017.00053

**Published:** 2017-03-28

**Authors:** Lisa S. Robison, Michalis Michaelos, Jason Gandhi, Dennis Fricke, Erick Miao, Chiu-Yim Lam, Anthony Mauceri, Melissa Vitale, Junho Lee, Soyeh Paeng, David E. Komatsu, Michael Hadjiargyrou, Panayotis K. Thanos

**Affiliations:** ^1^Department of Psychology, Stony Brook UniversityStony Brook, NY, USA; ^2^Research Institute on Addictions, University at BuffaloBuffalo, NY, USA; ^3^Department of Orthopedics, Stony Brook UniversityStony Brook, NY, USA; ^4^Department of Life Sciences, New York Institute of TechnologyOld Westbury, NY, USA

**Keywords:** methylphenidate, ritalin, psychostimulant, attention deficit hyperactivity disorder, sensitization, sex differences

## Abstract

Methylphenidate (MP) is a psychostimulant prescribed for Attention Deficit Hyperactivity Disorder. Previously, we developed a dual bottle 8-h-limited-access-drinking-paradigm for oral MP treatment of rats that mimics the pharmacokinetic profile of treated patients. This study assessed sex differences in response to this treatment. Male and female Sprague Dawley rats were assigned to one of three treatment groups at 4 weeks of age (*n* = 12/group): Control (water), low dose (LD) MP, and high dose (HD) MP. Rats drank 4 mg/kg MP (LD) or 30 mg/kg MP (HD) during the first hour, and 10 mg/kg (LD) or 60 mg/kg MP (HD) for the remaining 7 h each day. Throughout 3 months of treatment, rats were monitored for body weight, food intake, and fluid intake; as well as tested for open field behavior, circadian activity, novel object recognition, and social interaction. Chronic MP treated rats exhibited reduced fluid intake during distinct treatment weeks to a greater extent in males, and reduced total fluid intake in males only. HD MP treatment decreased body weight in both sexes, while HD MP increased total food intake in females only, likely to offset energy deficits resulting from MP-induced hyperactivity. LD and HD MP increased locomotor activity in the open field, particularly in females and during later treatment weeks. MP dose-dependently increased activity during the dark cycle of circadian testing in females, while in males hyperactivity was only exhibited by HD rats. HD MP increased center activity to a greater extent in males, while MP increased rearing behavior in females only. MP had no effect on social behavior or novel object recognition in either sex. This study concludes that chronic oral MP treatment at clinically-relevant dosages has significant effects on food intake, body weight, open field behavior, and wake cycle activity. Particularly marked sex differences were apparent for locomotor activity, with females being significantly more sensitive to the hyperactivating effects of the drug. These findings suggest that chronic MP exposure beginning in adolescence can have significant behavioral effects that are both dose- and sex-dependent, and raise concerns regarding the reversibility of these effects post-discontinuation of treatment.

## Introduction

Attention Deficit Hyperactivity Disorder (ADHD), with typical symptoms of inattention, hyperactivity, and impulsivity beginning in childhood, is one of the most frequently diagnosed neuropsychiatric disorders. Diagnosis rates of ADHD have jumped to ~11% of school-aged children in the United States, an increase of over 40% during the last decade (Visser et al., 2014). Approximately two-thirds of these individuals are treated with psychostimulants such as methylphenidate (MP), which are also used illicitly as a study aid among high school and college students and abused recreationally (McCabe et al., 2006; Teter et al., 2006; Wilens et al., 2008). Concern has arisen about the use of MP during critical periods of neurodevelopment, such as adolescence, when the brain is particularly susceptible to external stimuli (Spear, 2000; Dahl, 2004). During this stage of development, the brain undergoes numerous changes in regions such as the prefrontal cortex, hippocampus, and limbic system, including the sprouting and pruning of synapses and changes in neurotransmitter concentrations and receptor levels (Rice and Barone, 2000; Spear, 2000; Dahl, 2004; Giedd, 2005). This presents concerns regarding subsequent effects of MP on neurobiology, development, and behavior.

In rodents, it has been shown that MP treatment results in alterations in neurobiology and several types of behavior, including locomotion, emotional behaviors, cognition, memory, and responses to natural and drug rewards (Wultz et al., 1990; Mueller, 1993; Gaytan et al., 1996, 2000; Izenwasser et al., 1999; Brandon et al., 2001; Heyser et al., 2004; Arnsten and Dudley, 2005; Mague et al., 2005; Berridge et al., 2006; Chuhan and Taukulis, 2006; Gray et al., 2007; Thanos et al., 2007; Zhu et al., 2010). A few major caveats exist, however, regarding a majority of these prior studies. These include issues with treatment procedures (treatment length and route of administration/dosing regimen), and a lack of assessment of sex differences in response to the drug.

Most previous studies on the effects of MP provide treatment for a few weeks or less. It is estimated that 50–60% of children and adolescents diagnosed with ADHD have symptoms that persist beyond childhood, prolonging MP treatment into adulthood (Mick et al., 2004; Faraone et al., 2008). This statistic compels research using longer treatment regimens in animals as well. Moreover, past studies in rodents have utilized an MP dosing regimen that does not correspond to clinical dosing (oral doses of 0.25–1 mg/kg MP, resulting in plasma concentrations of 8–40 ng/mL) (Swanson et al., 1999; Swanson and Volkow, 2002). Several of these studies injected MP either subcutaneously or intraperitoneally, which differs significantly from oral administration, specifically with respect to time to peak serum concentration, half-life, and rate of elimination (Kuczenski and Segal, 2001), as well as absolute magnitude and time course of increases in extracellular DA and locomotor responses (Gerasimov et al., 2000; Kuczenski and Segal, 2001). Studies that have used oral dosing have done so by gavage (Kuczenski and Segal, 2001; Justo et al., 2010), which is stressful and can cause injury (Brown et al., 2000; Balcombe et al., 2004), or by administering MP on an oyster cracker or by mixing with chow (LeBlanc-Duchin and Taukulis, 2007; Zhu et al., 2010). Although the latter is less stressful and dangerous, oral administration results in peak serum concentration 15 min post-administration, and this concentration has been shown to drop by half within an additional 5 min (Patrick et al., 1984). The faster metabolism and shorter half-life of MP in rats compared to humans would therefore necessitate nearly constant dosing to maintain clinically-relevant plasma concentrations, unlike previous studies that dosed animals only once or twice per day (Kuczenski and Segal, 2001; LeBlanc-Duchin and Taukulis, 2007; Justo et al., 2010; Zhu et al., 2010).

Lastly, most prior studies of MP in animal models have utilized males only. Although the historical lack of inclusion of female subjects in scientific research is unfortunately common, this has also been justified by the apparent gender bias in ADHD, with lifetime diagnosis rates of males being nearly double that of females (Bloom et al., 2012). It has been suggested that differences in diagnosis rates may be at least in part due to differences in manifestations of symptoms. Whereas, males may exhibit more observable behavioral issues (hyperactivity and impulsivity), females may struggle more silently with cognitive dysfunction (inattention) (Gaub and Carlson, 1997; Gershon and Gershon, 2002; Biederman et al., 2005). This discovery will likely increase ADHD diagnosis and subsequent treatment in females in the coming years, further supporting the need for research on possible sex differences in response to psychostimulant treatment. It has been well-documented that females are subject to different responses to drug treatments due to sex-specific pharmacological signaling differences, (i.e., growth development and hormonal distribution) (Brown et al., 2000; Zakharova et al., 2009; Tingen et al., 2010), and have been shown to be more sensitive to some of the effects of psychostimulants (Walker et al., 2001; Carrier and Kabbaj, 2012; Chelaru et al., 2012; Van Swearingen et al., 2013).

Previously, we developed a dual-bottle 8 h limited-access drinking paradigm that allowed MP to be consumed voluntarily in the rats' drinking water (Thanos et al., 2015). Briefly, In addition to a water control group, two 8-h-limited-access dosing regimens were used created: 4 mg/kg MP (low dose; LD) or 30 mg/kg MP (high dose; HD) during the first hour, and 10 mg/kg (LD) or 60 mg/kg (HD) MP from hours two through eight. In rats, these oral doses were shown to produce plasma MP concentrations stably within the clinical pharmacokinetic range (peak at ~8 ng/mL for the LD and ~30 ng/mL for the HD) for an eight to 10 h period (Thanos et al., 2015). Three months of MP treatment in male rats starting in adolescence resulted in substantially altered body weight, food intake, open field behavior, and circadian activity (Thanos et al., 2015). This study, however, did not examine females to assess possible sex differences in response to clinically-relevant doses of MP. In the current study, male and female rats chronically treated with MP beginning in adolescence were tested for developmental parameters (body weight, food intake), as well as behavior (open field locomotor behavior, circadian locomotor activity, social behavior), and cognitive function (novel object recognition). We hypothesized that chronic MP treatment would result in sexually dimorphic effects on physiology and behavior.

## Materials and methods

### Animals

Male (*n* = 36) and female (*n* = 36) 4 week old Sprague Dawley rats (Taconic, Hudson, New York USA) were individually housed in a controlled room (22 ± 2°C and 40–60% humidity) with a 12 h reverse light-dark cycle (lights off 0800 h). Rats of each sex were split into three groups (*n* = 12/group): Control (drinking only water), low dose MP (LD), or high dose MP (HD). Rats were treated for 3 months using a previously established dual-bottle 8-hour limited access drinking paradigm (Thanos et al., 2015). Rats received 4 mg/kg MP (LD) or 30 mg/kg MP (HD) during the first hour (09:00–10:00 h), and 10 mg/kg (LD) or 60 mg/kg MP (HD) for the remaining 7 h (10:00–17:00 h). Purina Lab Diet rat chow was available *ad libitum* for the entire experiment, and body weight, food intake, and fluid intake were recorded daily. All experiments were conducted in conformity with the National Academy of Sciences Guide for Care and Use of Laboratory Animals and approved by the Stony Brook University Institutional Animal Care and Use Committee.

### Drugs

Methylphenidate hydrochloride (Sigma Aldrich, St. Louis, MO) was dissolved in distilled water to produce the 4, 10, 30, and 60 mg/kg solutions for each rat individually, based on body weight and the average volume of fluid consumed over the previous 3 days.

### Procedures

#### Open field test

Animals were run in an open-field arena photo beam activity monitoring system (Coulbourn Instruments, Allentown, PA) for 90 min to test locomotor activity prior to treatment and once per week throughout the treatment period. Tests were performed between 11:00 and 17:00 h. Open field locomotor data was acquired with Tru Scan v2.0 software, and activity measures recorded include: (a) distance traveled; (b) velocity; (c) relative center distance (distance traveled in the center/distance traveled in the periphery); (d) center time; (e) rearing events; and (f) rearing time.

#### Circadian activity

Rats were tested for circadian locomotor activity over 24 h during the last week of chronic MP treatment, measured by an optical beam sensor over their home cages (Minimitter Vital View software; Bend, Oregon), as has been done in previous studies assessing behavioral effects of methylphenidate (Thanos et al., 2015). Beam breaks were recorded and binned by minute, then summed in two ways: (1) total number of beam breaks per hour, and (2) total number of beam breaks in the dark (08:00–20:00 h) and light (20:00–08:00 h) cycles. Throughout circadian activity testing, food was provided *ad libitum*, and the 8 h limited access drinking paradigm was kept in place, with rats having normal access to water or respective MP solution.

#### Social interaction

The social interaction test was performed during the last week of treatment. Testing procedures were performed in a dimly illuminated room during the dark cycle between 11:00 and 17:00 h. The social interaction test was conducted in the same arenas as the weekly locomotor tests. Experimental rats were first placed in the arenas alone for 5 min for habituation. For female subjects, a non-experimental female conspecific rat of similar age and body weight was then placed into the arena with the experimental rat for 5 min. For male subjects, a 3 week old non-experimental male rat was placed into the arena with the experimental rat for 5 min. As in a previous study (Lukas et al., 2011), a juvenile male rat was chosen as a social stimulus because it does not elicit aggressive behavior by adult male rats. Arenas were thoroughly wiped down with 10% ammonia between runs to avoid the influence of olfactory cues from previous rats. Each run was recorded and later rated by two experimenters blind to treatment condition. Time spent engaging in active social interaction (i.e., grooming, sniffing, following, climbing over, wrestling/boxing) was recorded for each experimental rat.

#### Novel object recognition

The novel object recognition (NOR) test assessed the rats' ability to recognize and distinguish between objects during the last week of treatment (Ennaceur and Delacour, 1988). Tests were performed in a dimly illuminated room during the dark cycle between 11:00 and 17:00 h. The NOR test was conducted in the same arenas as the weekly locomotor tests, eliminating the need for habituation to the environment. The NOR test was set up by placing two identical objects oriented in diagonal corners of the arena, with each 6 cm from the wall of the arena. Rats were first placed in the NOR arena for this 5 min acquisition run. Following a 30 min break in the home cage, rats were put back in the arena for the 5 min retention run, in which a novel object replaced one of the initial (i.e., familiar) objects. Arenas and objects were thoroughly wiped down with 10% ammonia between runs to avoid the influence of olfactory cues from previous rats. Each run was recorded and time spent exploring each object was rated by two raters that were blind to the treatment that each rat received. Exploration was measured when a rat's nose physically touched, sniffed, or approached an object to within two centimeters of the object. Discrimination was assessed for the retention run using the discrimination index [DI = (time spent exploring novel object/total exploration time)–(time spent exploring familiar object/total exploration time)].

#### Statistical analysis

Body weight, food intake, fluid intake, and open field locomotor activity data were analyzed using three-way repeated measures ANOVAs [between-subjects factors: Treatment (water, LD MP, or HD MP) and sex; within-subjects factor: Time (week of treatment)]. Three-way repeated measures ANOVAs were also run to analyze circadian activity data [between-subjects factor: Treatment and sex; within-subjects factor: Time (hour of day or light cycle)]. A three-way repeated measures ANOVA [between-subjects factors: Treatment (water, LD MP, or HD MP) and sex; within-subjects factor: Object (familiar or novel)] was used to assess time spent interacting with objects in the novel object recognition test. Two-way ANOVAs (between-subjects factors: Treatment and sex) were used to analyze data from the novel object recognition (discrimination index) and social interaction tests, as well as total fluid consumption, total food consumption, and percent change in body weight from pretreatment to the final week of treatment. When appropriate, follow-up pair-wise comparisons were performed using the Tukey method. Statistical significance was set at α = 0.05 for all tests. Two-way ANOVA statistical tests were run using SigmaPlot 11.0 software, and three-way repeated measures ANOVA statistical tests were run using Statistica 8.0 software.

## Results

### Fluid intake

**Figure 1:**
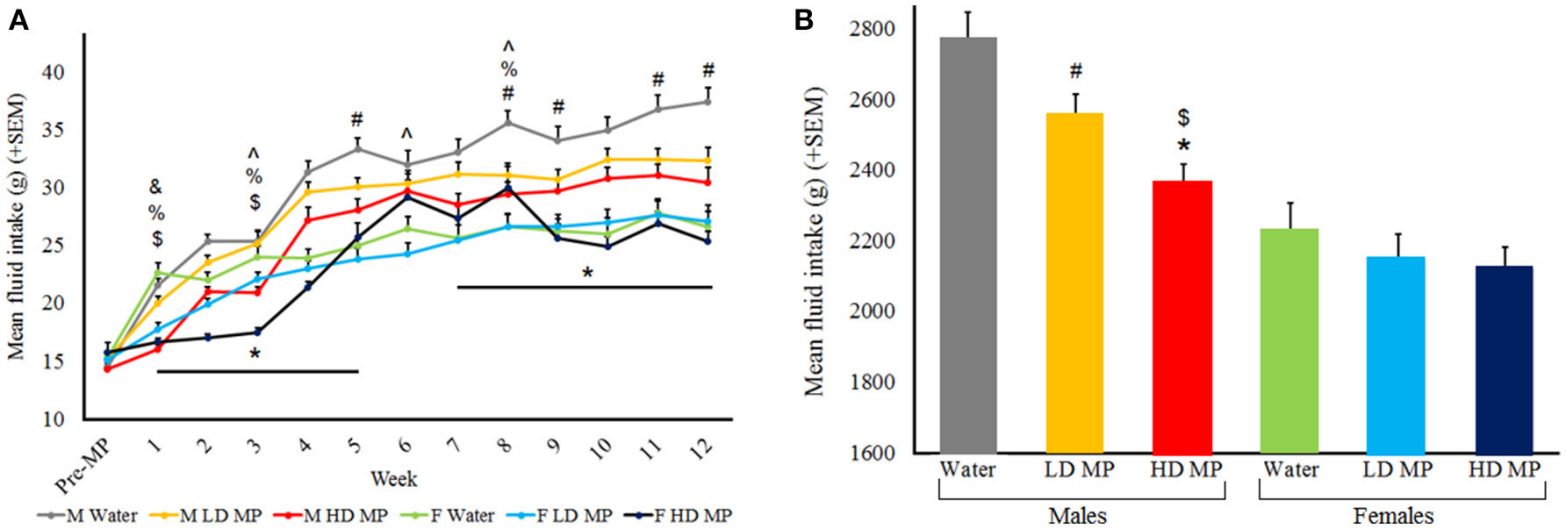
**(A)** Mean (+SEM) daily fluid intake by treatment week. All groups generally increased fluid intake as they grew from adolescents into adults. Males had greater fluid intake than females. HD male rats drank less than water control rats during treatment weeks 1–5 and 7–12 (^*^p < 0.05); HD male rats also drank less than LD rats in treatment weeks 1 and 3 (^$^*p* < 0.05); LD male rats drank less than water control rats for treatment weeks 5, 8, 9, 11, and 12 (^#^*p* < 0.05). HD female rats drank less than water control rats in treatment week 1, 3, and 8 (^%^*p* < 0.05). HD female rats drank less than LD female rats in week 3, but drank more in weeks 6 and 8 (^∧^*p* < 0.05). LD female rats drank less than water control females in week 1 (^&^*p* < 0.05). **(B)** Mean (+SEM) total fluid consumption throughout the entire experiment. HD MP males drank less than both LD MP (^$^*p* < 0.05) and water (^*^p < 0.05) treated males, while LD MP males drank less than water treated males (^#^*p* < 0.05).

Fluid intake was measured daily and averaged weekly for each treatment group (Figure 1A). A three-way repeated measures ANOVA revealed that the main effects of treatment [*F*_(2, 66)_ = 6.49, *p* < 0.01], sex [*F*_(1, 66)_ = 47.37, *p* < 0.001], and time [*F*_(12, 792)_ = 256.31, *p* < 0.001] were significant on fluid intake. The sex × time [*F*_(12, 792)_ = 17.08, *p* < 0.001], and treatment × time [*F*_(24, 792)_ = 4.20, *p* < 0.001] interactions were also significant, and the treatment × sex interaction was only marginally significant [*F*_(2, 66)_ = 2.94, *p* = 0.060]. Results of pairwise comparisons can be seen in Table 1. Additionally, the treatment × sex × time interaction was significant [*F*_(24, 792)_ = 3.36, *p* < 0.001]. Male HD MP rats showed decreased fluid intake in comparison to male water control rats throughout treatment, as well as decreased consumption compared to LD MP rats in early treatment weeks. Male LD MP rats showed decreased fluid intake in comparison to male water control rats in later treatment weeks. Female HD MP rats showed decreased fluid consumption in comparison to LD MP and water control female rats in early treatment weeks; however, fluid consumption in female HD rats was higher than both other groups in middle treatment weeks. Female LD MP rats only showed significantly decreased fluid intake, compared to water control females, in the first treatment week. Specific pairwise comparisons can be seen in Table 1.

**Table 1:** **Pairwise comparisons of significant main effects of drug and sex, and their interactions over time**.

	**Drug**	**Sex**	**Drug × Sex**	**Drug × Time**	**Drug × Sex × Time**
Fluid intake	HD & LD < Water	M > F	M > F (all treatment groups)	HD < Water (1–5, 9–12)	M: HD < Water (1–5, 7–12)
			M: HD & LD < Water	HD < LD (1, 3–5)	M: LD < Water (5, 8, 9, 11, 12)
				LD < Water (1, 5, 8, 11, 12)	M: HD < LD (1, 3)
					F: HD < Water (1, 3)
					F: HD > Water (8)
					F: LD < Water (1)
					F: HD < LD (3)
					F: HD > LD (6, 8)
Food intake	LD > Water	M > F	M > F (all treatment groups)	HD < Water (1, 3)	M: HD < Water (1)
			F: HD > Water	HD > Water (9–12)	M: HD < LD (1–5)
				HD < LD (pre-MP, 1, 3, 4)	M: LD > Water (3–5)
				HD > LD (12)	F: HD > Water (2, 6, 8–12)
				LD > Water (pre-MP, 2, 4, 9, 10)	F: HD < Water (3)
					F: HD > LD (1, 3, 8–12)
					F: LD > Water (9, 10)
Body weight	HD < Water & LD	M > F	M > F (all treatment groups)	HD < LD & Water (1–12)	M: HD < Water (1–12)
			HD < LD & Water (M & F)		M: HD < LD (2–12)
					F: HD < Water (4–12)
					F: HD < LD (3–12)
Open field: Distance	HD > LD > Water	F > M	M: HD > Water & LD	HD > Water (1–12)	M: HD > LD & Water (6, 8-12)
			F: HD & LD > Water	HD > LD (2–12)	F: HD > Water (1–12)
			LD & HD: F > M	LD > Water (8–12)	F: LD > Water (1, 5, 7–12)
					F: HD > LD (2–12)
					HD: F > M (2–12)
					LD: F > M (6–12)
Open field: Velocity	HD > LD > Water	F > M	M: HD > Water & LD	HD > Water (1–12)	M: HD > Water (6, 8–12)
			F: HD > Water & LD	HD > LD (2–12)	M: HD > LD (6–8, 10–12)
			LD: F > M	LD > Water (8–12)	F: HD > Water (1–12)
			HD: F > M		F: LD > Water (5, 7–12)
					F: HD > LD (2–12)
					F: LD < Water (1)
					HD: F > M (2–12)
					LD: F < M (1)
					LD: F > M (5–12)
Open field: Relative center distance	HD > Water & LD	M > F	HD > Water & LD (M)		
			M > F (all treatment groups)		
Open field: Center time	HD > Water & LD	M > F			
Open field: Rearing events	HD > Water & LD	F > M	HD > Water & LD (M & F)	HD > Water (1-12)	
			LD: F > M	LD > Water (5, 7)	
			HD: F > M	HD > LD (2–12)	
			F: LD > Water		
Open field: Rearing time	HD & LD > Water	F > M	F > M (all drug treatment groups)	HD > Water (2–5, 7–12)	
			F: HD & LD > Water	HD > LD (4)	
				HD < LD (12)	
				LD > Water (5, 7–9, 11-12)	
Circadian locomotor	HD > LD > Water	F > M		HD > Water (10–18 h)	M: HD > Water (16 h)
Activity (Hourly)				HD > LD (11, 14–16 h)	F: HD > Water (10–18 h), F: HD > LD (14–18 h)
					HD: F > M (17–18 h)
Circadian locomotor	HD > LD > Water	F > M		HD > LD > Water (dark cycle)	M: HD > Water (dark cycle)
Activity (by Cycle)					M: HD > LD (dark cycle)
					F: HD > Water (dark cycle)
					F: HD > LD (dark cycle)
					F: LD > Water (dark cycle)
					HD: F > M (dark cycle)

A two-way ANOVA was run for total fluid consumption throughout the entire experiment (Figure 1B). The main effects of treatment [*F*_(2, 66)_ = 6.488, *p* < 0.01] and sex [*F*_(1, 66)_ = 47.374, *p* < 0.001] were significant, such that males drank more than females (*p* < 0.001) and water treated rats drank more than HD (*p* < 0.001) and LD (*p* < 0.05) MP treated rats. The treatment × sex interaction approached significance [*F*_(2, 66)_ = 2.944, *p* = 0.060], such that HD MP males drank less than both LD MP and water treated males, while LD MP males drank less than water treated males (*p* < 0.05 for all) There were no group differences in females.

### Food intake

**Figure 2:**
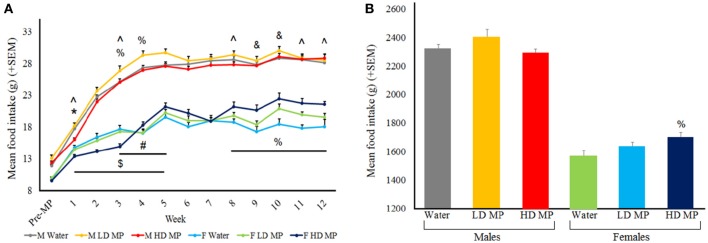
**(A)** Mean (+SEM) daily food intake by treatment week. Rats expectedly increased food intake as they grew from adolescents into adults. Males had greater food intake in comparison to females within all groups of treatment. HD male rats ate less than water control males rats during the first treatment week (^*^p < 0.05), and ate less than LD rats in treatment weeks 1–5 (^$^*p* < 0.05). LD male rats ate more than water control rats for treatment weeks 3–5 (^#^*p* < 0.05). HD female rats ate less than water control female rats in treatment week 3, but ate more in comparison of treatment week 4, 8–12 (^%^*p* < 0.05) HD female rats ate less than LD female rats during treatment weeks 1, 3, 8, 11, and 12 (^∧^*p* < 0.05). LD female rats ate more than water control females during treatment weeks 9 and 10 (^&^*p* < 0.05). **(B)** Mean (+SEM) total food consumption throughout the entire experiment, with HD MP females eating more than water treated females (^%^*p* < 0.05).

Food intake was measured daily and averaged weekly for each treatment group (Figure 2A). A three-way repeated measures ANOVA found that the main effects of sex [*F*_(1, 66)_ = 434.57, *p* < 0.001] and time [*F*_(12, 792)_ = 556.17, *p* < 0.001] on food intake were significant, while the main effect of treatment on food intake was marginally significant [*F*_(2, 66)_ = 2.63, *p* = 0.079]. The sex × time [*F*_(12, 792)_ = 61.33, *p* < 0.001] and treatment × time [*F*_(24, 792)_ = 5.72, *p* < 0.001] interactions were significant, while the treatment × sex interaction was marginally significant [*F*_(2, 66)_ = 2.64, *p* = 0.078]. Results of pairwise comparisons can be seen in Table 1. Additionally, the treatment × sex × time interaction was significant [*F*_(24, 792)_ = 3.88, *p* < 0.001]. HD MP males showed decreased food intake compared to water controls and LD MP males during early treatment weeks. LD MP males had greater food intake compared to water control males in early to mid-treatment weeks. HD MP females had significantly increased food intake compared to water controls and LD MP females in some early to mid, and consistently later, treatment weeks. LD MP females consumed significantly more food than water control females during later treatment weeks. Specific pairwise comparisons can be seen in Table 1.

A two-way ANOVA was run for total food consumption throughout the entire experiment (Figure 2B). The main effect of sex was significant [*F*_(1, 66)_ = 435.734, *p* < 0.001], such that males ate more than females (*p* < 0.001), while the main effect of treatment was not significant [*F*_(2, 66)_ = 2.087, *p* = 0.132]. The treatment × sex interaction approached significance [*F*_(2, 66)_ = 2.955, *p* = 0.059], such that HD MP females ate more than water treated females (*p* < 0.05). There were no overall differences in total food intake between treatment groups in males.

### Body weight

**Figure 3:**
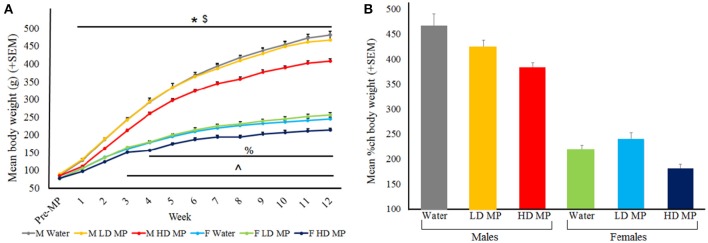
**(A)** Mean (+SEM) body weight by treatment week. Rats expectedly gained weight as they grew from adolescents into adults. Males expectedly gained more weight than females. HD MP treatment dose-dependently attenuated body weight throughout most of the treatment period. HD male rats weighed significantly less than water control males (^*^p < 0.05) and LD males (^$^*p* < 0.05) in treatment weeks 1–12. HD MP female rats weighed less than water control females in treatment weeks 4–12 (^%^*p* < 0.05) and less than LD MP female rats in treatment weeks 3–12 (^∧^*p* < 0.05). **(B)** Mean (+SEM) percent change in body weight from pretreatment to the final week of the experiment. HD MP attenuated weight gain, regardless of sex.

Body weight was measured daily and averaged weekly for each treatment group (Figure 3). A three-way repeated measures ANOVA found that the main effects of treatment [*F*_(2, 66)_ = 47.44, *p* < 0.001], time [*F*_(12, 792)_ = 5597.07, *p* < 0.001], and sex [*F*_(1, 66)_ = 1572.94, *p* < 0.001] on body weight were all significant, as were the treatment × sex [*F*_(2, 66)_ = 5.21, *p* < 0.01], sex × time [*F*_(12, 792_ = 979.83, *p* < 0.001], and treatment × time [*F*_(24, 792)_ = 23.48, *p* < 0.001] interactions. Results of pairwise comparisons can be seen in Table 1. There was also a significant treatment × sex × time interaction [*F*_(24, 792)_ = 2.59, *p* < 0.001]. While HD MP treatment reduced body weight compared to water treatment in males throughout all treatment weeks; this effect took longer to appear in females (starting at week 4). HD MP rats of both sexes also had reduced body weight in comparison to LD MP rats in all but the first few weeks of treatment. Specific pairwise comparisons can be seen in Table 1.

A two-way ANOVA was run for percent change in body weight between the pretreatment period and the final MP treatment week (Figure 3B). The main effect of treatment was significant [*F*_(2, 66)_ = 9.025, *p* < 0.001], with HD MP rats gaining less weight than both water (*p* < 0.001) and LD MP treated rats (*p* < 0.01). The main effect of sex was also significant [*F*_(1, 66)_ = 284.005, *p* < 0.001], with males gaining more weight than females throughout the course of the experiment (*p* < 0.001). The treatment × sex interaction was not significant (*p* > 0.05).

### Open field

Rats were run in an open field arena once prior to treatment and once per week during treatment, assessing horizontal activity (distance traveled and velocity), center activity (relative center distance and center time), and rearing activity (rearing events and rearing time).

#### Horizontal activity

**Figure 4:**
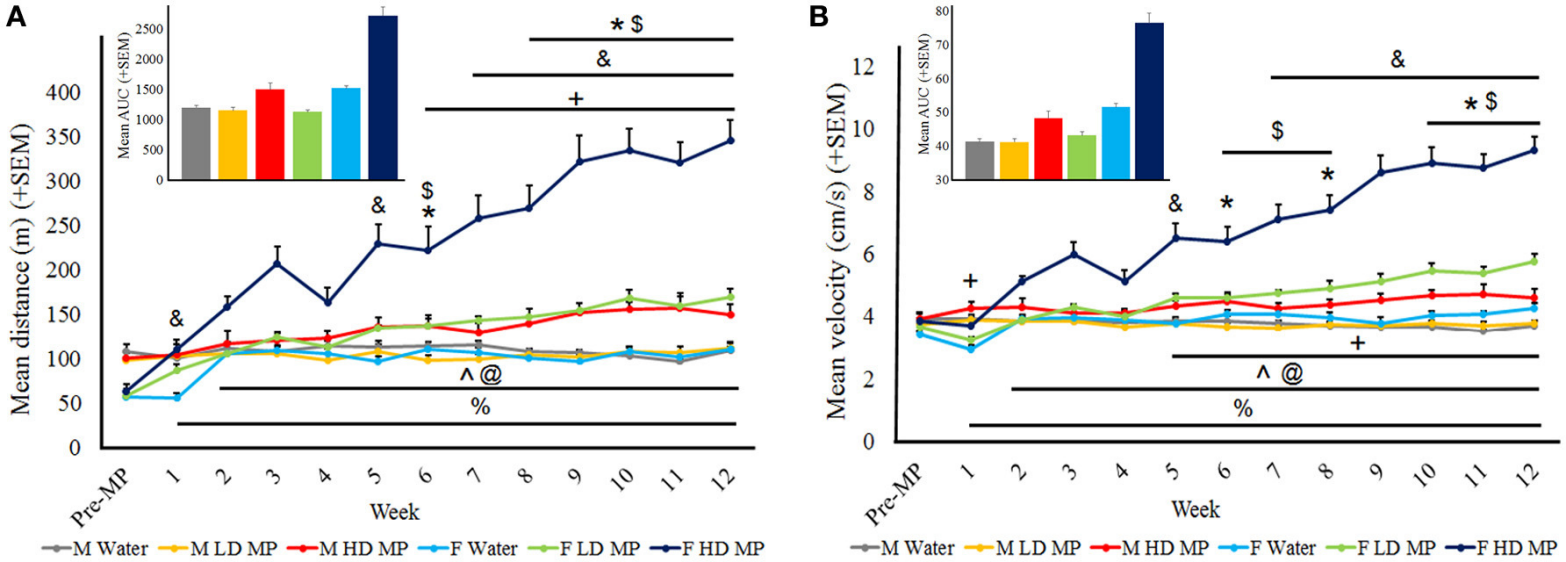
**Horizontal activity in the open field. (A)** Mean (+SEM) distance traveled in the open field. There was an overall increase in distance traveled as time passed. Male HD MP rats exhibited greater activity than male water (^*^p < 0.05) and LD MP (^$^*p* < 0.05) rats in weeks 6 and 8–12. Female LD MP rats traveled a greater distance than female water rats in weeks 1, 5, 7–12 (^&^*p* < 0.05) while female HD MP rats were more active than water rats in all MP treatment weeks (%*p* < 0.001). Female HD MP rats were also more active than female LD MP rats in weeks 2–12 (^∧^*p* < 0.001). Females were more active than males on HD (weeks 2–12, ^@^*p* < 0.05) and LD (weeks 6–12, +*p* < 0.05) MP treatment. Insert graph shows area under the curve by treatment group for all treatment weeks. **(B)** Mean (+SEM) velocity in the open field. There was an overall increase in velocity as time passed. Male HD MP rats had greater velocity than water rats in weeks 6, 8, and 10–12 (^*^p < 0.05) and greater velocity than LD MP rats in weeks 6–8 and 10–12 (^$^*p* < 0.05). Female LD MP rats had significantly greater velocity than female water rats in weeks 5 and 7–12 (^&^*p* < 0.05). Female HD MP rats had significantly greater velocity than female water rats in all MP treatment weeks (^%^*p* < 0.001) and also greater than female LD MP rats in weeks 2–12 (^∧^*p* < 0.001). Females moved at a greater velocity than males on HD (weeks 2–12, ^@^*p* < 0.05) and LD (weeks 5–12, ^+^*p* < 0.05) MP treatment, although LD MP females moved at a slower velocity water treated females in week 1 (^&^*p* < 0.05). Insert graph shows area under the curve by treatment group for all treatment weeks.

Distance traveled data was averaged within treatment groups for each week (Figure 4A). A three-way repeated measures ANOVA found that the main effects of treatment [*F*_(2, 66)_ = 81.219; *p* < 0.001], sex [*F*_(1, 66)_ = 52.043; *p* < 0.001], and time [*F*_(12, 792)_ = 45.72; *p* < 0.001] on distance were significant, as were the treatment × sex [*F*_(2, 66)_ = 36.143; *p* < 0.001], sex × time [*F*_(12, 792)_ = 26.334; *p* < 0.001] and treatment × time × interactions [*F*_(24, 792)_ = 14.685; *p* < 0.001]. Results of pairwise comparisons can be seen in Table 1. Additionally, there was a significant treatment × sex × time interaction [*F*_(24, 792)_ = 5.686; *p* < 0.001]. While HD MP treatment increased distance compared to water treatment in females throughout all treatment weeks, this treatment effect took longer to appear in males (starting at week 6). Female HD MP rats also exhibited greater distance than female LD MP and male HD MP rats in all but the first week of treatment. Female LD MP rats had significantly more distance than female water rats in most treatment weeks, with this effect being more pronounced during later treatment weeks. Female LD MP rats exhibited greater distance than male LD MP rats in mid to late treatment. Specific pairwise comparisons can be seen in Table 1.

Velocity was averaged within each treatment group for each treatment week (Figure 4B). A three-way repeated measures ANOVA reported that the main effects of treatment [*F*_(2, 66)_ = 78.663; *p* < 0.001], sex [*F*_(1, 66)_ = 89.178; *p* < 0.001], and time [*F*_(12, 792)_ = 42.238; *p* < 0.001] were significant on FP velocity, as were the treatment × sex [*F*_(2, 66)_ = 31.506; *p* < 0.001], sex × time [*F*_(12, 792)_ = 40.299; *p* < 0.001], and treatment × time [*F*_(24, 792)_ = 14.584; *p* < 0.001] interactions. Results of pairwise comparisons can be seen in Table 1. There was also a significant treatment × sex x time interaction on velocity [*F*_(24, 792)_ = 7.793; *p* < 0.001]. Female HD MP rats had significantly greater velocity than female water rats in all treatment weeks and also greater than female LD MP rats in all but the first week of treatment. Female LD MP rats had significantly higher velocity than female water rats in mid to late treatment weeks. Male HD MP rats had greater velocity than water control and LD MP male rats in mid to late treatment weeks. Female HD MP rats had greater velocity than male HD MP rats in all but the first week of treatment, while female LD MP rats had greater velocity than male LD MP rats in mid to late treatment weeks. Specific pairwise comparisons can be seen in Table 1.

#### Center activity

**Figure 5:**
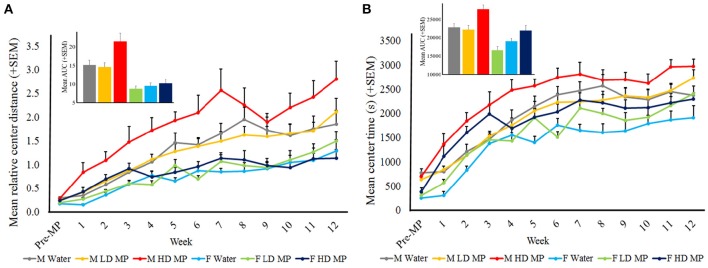
**Center activity in the open field. (A)** Mean (+SEM) relative center distance. There was an overall increase in center activity as time passed, and over time males exhibited greater center activity than females. HD MP treatment increased center activity compared to water and LD MP rats in males only. Insert graph shows area under the curve by treatment group for all treatment weeks. **(B)** Mean (+SEM) center time in the open field. There was an overall increase in center time as time passed. Overall, HD MP rats spent more time in the center of the arena than water and LD MP treated rats. Additionally, males exhibited more center time than females. Insert graph shows area under the curve by treatment group for all treatment weeks.

Open field runs also assessed center activity. Relative center distance was averaged within treatment groups for each week (Figure 5A). A three-way repeated measure ANOVA reported that the main effects of treatment [*F*_(2, 66)_ = 5.1749; *p* < 0.01], sex [*F*_(1, 66)_ = 50.6333, *p* < 0.001], and time [*F*_(12, 792)_ = 57.0294; *p* < 0.001] were significant, as were the treatment × sex [*F*_(2, 66)_ = 0.7528; *p* < 0.05] and sex × time [*F*_(12, 792)_ = 5.8290; *p* < 0.001] interactions. Male HD MP rats exhibited greater center activity than male water and LD MP treated rats (*p* ≤ 0.01 for both); there were no differences between treatment groups in females (p > 0.05). The treatment × time and treatment × sex × time interactions were not significant (*p* > 0.05). All pairwise comparisons for significant main effects and interactions can be seen in Table 1.

Center time was averaged within treatment groups for each week (Figure 5B). A three-way repeated measure ANOVA revealed a significant main effect of treatment on center time [*F*_(2, 66)_ = 13.125; *p* < 0.001], such that HD MP rats spent more time in the center of the arena than water or LD MP rats. The main effects of sex [*F*_(1, 66)_ = 36.391; *p* < 0.001] and time [*F*_(12, 792)_ = 112.330; *p* < 0.001] were also significant, as was the sex × time interaction [*F*_(12, 792)_ = 1.907; *p* < 0.05)]. The treatment × sex, treatment × time, and treatment × sex × time interactions had no significant effect on center time. Results of pairwise comparisons can be seen in Table 1.

#### Rearing activity

**Figure 6:**
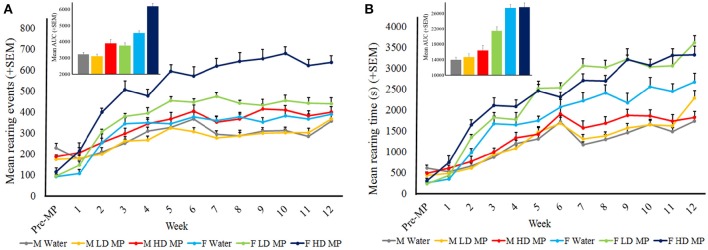
**Rearing activity in the open field. (A)** Mean (+SEM) rearing events in the open field. There was an overall increase in rearing events as time passed. HD rats exhibited more rearing events than both water and LD MP treated rats in both males and females, and throughout most treatment weeks. LD MP increased rearing events in females only. LD and HD MP treated females exhibited greater rearing events than their male counterparts. Insert graph shows area under the curve by treatment group for all treatment weeks. **(B)** Mean (+SEM) rearing time in the open field. There was an overall increase in rearing time as time passed. Females of all treatment groups exhibited greater rearing time than their male counterparts. LD and HD MP increased rearing time in several weeks, however both MP doses increased rearing time overall in female rats only. Insert graph shows area under the curve by treatment group for all treatment weeks.

The weekly open field runs also measured rearing activity. Rearing events were averaged within treatment groups for each week (Figure 6A). A three-way repeated measures ANOVA reported that the main effects of treatment [*F*_(2, 66)_ = 45.847; *p* < 0.001)], sex [*F*_(1, 66)_ = 86.198; *p* < 0.001], and time [*F*_(12, 792)_ = 122.943; *p* < 0.001] were significant, as was the sex × time interaction [*F*_(12, 792)_ = 22.521; *p* < 0.001)]. Results of pairwise comparisons can be seen in Table 1. There was also a significant treatment × sex interaction on rearing events [*F*_(2, 66)_ = 14.410; *p* < 0.001], such that female LD and HD MP treated rats had significantly more rearing events than their male treated counterparts. Male and female HD MP rats had significantly more rearing events when compared to their water and LD MP counterparts. Female LD MP rats also had more rearing events than water-treated counterparts. The treatment x time interaction effect was also significant on rearing events [*F*_(24, 792)_ = 4.282; *p* < 0.001]. HD MP rats had significantly more rearing events than water and LD MP rats in almost all MP treatment weeks. LD MP rats had significantly more rearing events than water control rats in mid treatment weeks. Specific pairwise comparisons can be seen in Table 1. The treatment × sex × time interaction was not significant (*p* > 0.05).

Data on rearing time were averaged within treatment groups for each week (Figure 6B). A three-way repeated measures ANOVA reported that the main effects of treatment [*F*_(2, 66)_ = 10.041; *p* < 0.01], sex [*F*_(1, 66)_ = 151.907; *p* < 0.001], and time [*F*_(12, 792)_ = 227.980; *p* < 0.001] were significant, as was the sex × time interaction [*F*_(12, 792)_ = 30.986; *p* < 0.001)]. Results of pairwise comparisons can be seen in Table 1. Additionally, there was a significant treatment × sex interaction [*F*_(2, 66)_ = 3.805; *p* < 0.05]. Within females, HD and LD MP treated rats exhibited increased rearing time compared to water controls. Females of all treatment groups also showed increased vertical plane time compared to their male counterparts. The treatment × time interaction effect was also significant on rearing time [*F*_(24, 792)_ = 3.223; *p* < 0.001]. LD and HD MP treated rats displayed greater rearing time compared to water treated rats during mid to late treatment, and throughout treatment, respectively. There was no overall significant treatment × sex × time interaction on rearing time (*p* > 0.05).

### Circadian locomotor activity

**Figure 7:**
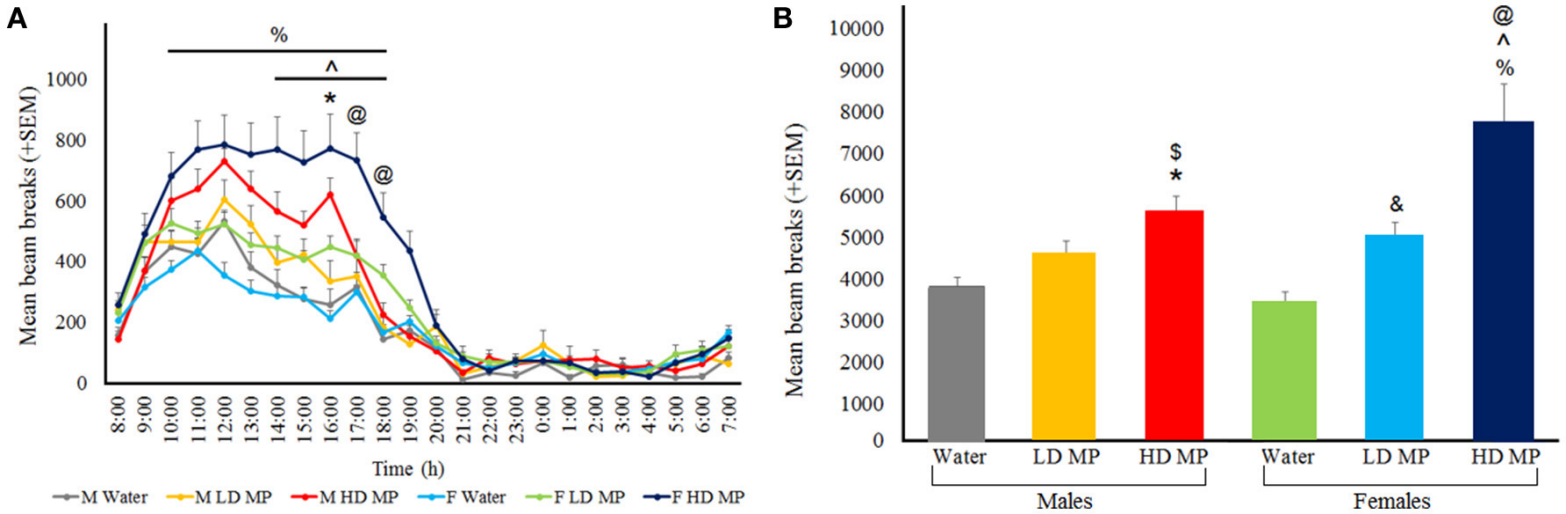
**Circadian locomotor activity during the last week of treatment. (A)** Mean (+SEM) hourly activity over the circadian cycle. A normal circadian cycle was exhibited by all groups, with no apparent shift in cycle. In females, HD MP treatment resulted in hyperactivity compared to both LD MP (^∧^*p* < 0.05) and water (^%^*p* < 0.05) treatment. HD males showed an increase in activity over water control males at 16:00 (^*^p < 0.05). HD females exhibited more activity in comparison to HD males at 17:00 to 18:00 (^@^*p* < 0.05). (B) Mean (+SEM) total activity throughout the dark cycle. In females, MP dose-dependently increased circadian activity [HD > Water (^%^*p* < 0.05); HD > LD (^∧^*p* < 0.05); LD > Water (^&^*p* < 0.05)], while in males HD MP increased activity compared to water (^*^p < 0.05) and LD MP ($*p* < 0.05) treated rats. Additionally, HD treated females were more active than male HD rats (^@^*p* < 0.05).

Circadian tests were run during the last week of treatment. Circadian hourly locomotor activity was measured and averaged within each treatment group of each sex (Figure 7A). A three-way repeated measures ANOVA found that the main effects of treatment [*F*_(2, 66)_ = 19.0563, *p* < 0.001], sex [*F*_(1, 66)_ = 4.2159, *p* < 0.05], and time [*F*_(23, 1518)_ = 188.1059, *p* < 0.001] were significant, as were the treatment × time [*F*_(46, 1518)_ = 9.9712, *p* < 0.001], and sex × time [*F*_(46, 1518)_ = 3.4860, *p* < 0.001] interactions. Results of pairwise comparisons can be seen in Table 1. There was also a significant treatment × sex × time interaction [*F*_(46, 1518)_ = 1.9680, *p* < 0.001]. Within males, HD MP rats were more active than water treated rats at only one timepoint in the middle of the dark cycle. Within females, HD MP rats were more active than water treated rats throughout most of the dark cycle following MP administration. HD MP females were also more active than LD MP females from the mid to late dark cycle. Within HD MP treated rats, females were more active than males during the last few hours of the dark cycle. Specific pairwise comparisons can be seen in Table 1.

Circadian light and dark cycle total activity levels were measured and averaged within each treatment group of each sex (Figure 7B). A three-way repeated measures ANOVA revealed that the main effects of treatment [*F*_(2, 66)_ = 19.0563, *p* < 0.001], sex [*F*_(1, 66)_ = 4.2159, *p* < 0.05], and cycle [*F*_(1, 66)_ = 544.1540, *p* < 0.001] were significant, as was the treatment × cycle interaction [*F*_(2, 66)_ = 21.9413, *p* < 0.001]. Results of pairwise comparisons can be seen in Table 1. The treatment × sex and sex × cycle interactions were not significant (*p* > 0.05). Additionally, there was a significant treatment × sex × cycle interaction [*F*_(2, 66)_ = 4.8121, *p* = 0.01]. LD MP rats were more active than water treated rats during the dark cycle; however, this was significant for females only (*p* < 0.01). HD MP rats were more active than water and LD MP treated rats during the dark cycle, and this was significant for both males and females (*p* < 0.05 for all). Within HD MP treated rats, females were more active than males during the dark cycle (*p* < 0.001). There were no differences between any groups during the light cycle (*p* > 0.05).

### Social interaction

Social interaction testing was conducted during the last week of treatment. The average time spent engaging in social interaction was calculated for each treatment group. A two-way ANOVA revealed that there was a significant main effect of sex [*F*_(1, 66)_ = 151.727, *p* < 0.001], with females spending more time interacting with their conspecific than males (*p* < 0.001). The main effect of treatment and the treatment × sex interaction were not significant (*p* > 0.05). Mean ± SEM for social interaction time by group: Male water (125.67 ± 10.65), Male LD MP (119.69 ± 9.86), Male HD MP (107.56 ± 9.19), Female water (232.75 ± 6.72), Female LD MP (234.08 ± 7.44), Female HD MP (219.33 ± 18.33).

### Novel object recognition

Novel object recognition testing was conducted during the last week of treatment. Time spent interacting with the familiar and novel objects was measured. A three way repeated measures ANOVA found a significant main effect of object [*F*_(1, 65)_ = 195.7753, *p* < 0.001], such that rats spent more time interacting with the novel object compared to the familiar object (*p* < 0.001). The main effect of sex was also significant [*F*_(1, 65)_ = 12.9599, *p* < 0.001], with males spending more time with objects overall than females (*p* < 0.001). The main effect of treatment and the treatment × sex, sex × object, treatment × object, and treatment × sex × object interactions were not significant (*p* > 0.05). Mean ± SEM time spent interacting with familiar object: Male water (20.86 ± 2.72), Male LD MP (25.56 ± 5.09), Male HD MP (17.36 ± 2.95), Female water (11.41 ± 1.65), Female LD MP (14.29 ± 1.46), Female HD MP (14.33 ± 2.66). Mean ± SEM time spent interacting with novel object: Male water (57.88 ± 5.01), Male LD MP (59.99 ± 7.70), Male HD MP (49.76 ± 4.50), Female water (42.41 ± 3.98), Female LD MP (49.29 ± 3.73), Female HD MP (46.33 ± 4.70).

The time spent interacting with objects was also used to determine the discrimination index, a ratio of time spent interacting with the novel vs. familiar object. A two-way ANOVA revealed that the effects of treatment, sex, and the treatment × sex interaction were not significant for novel object discrimination index (*p* > 0.05). Mean ± SEM for novel object discrimination index by group: Male water (46.13 ± 7.23), Male LD MP (47.06 ± 5.76), Male HD MP (50.64 ± 6.00), Female water (57.33 ± 5.01), Female LD MP (54.66 ± 4.18), Female HD MP (53.69 ± 7.42).

## Discussion

Due to the increasing trends of ADHD diagnosis and the use and abuse of psychostimulants, the current study aimed to determine the developmental and behavioral effects of chronic oral MP treatment in male and female Sprague Dawley rats (a non-ADHD model). Chronic treatment with MP, particularly with a clinically-relevant high dose, significantly impacted several measures including food intake, body weight, and locomotor behavior, with females being significantly more sensitive to the effects of MP compared to males, despite dosages being normalized by body weight. These findings have important clinical implications given the increasing rates of ADHD diagnosis and treatment and highlight special consideration that must be given to sex differences in response to being medicated with psychostimulants.

MP was found to decrease fluid intake in both sexes at distinct times of treatment. In males, HD treatment attenuated fluid intake throughout treatment, while LD treatment had this effect in later weeks of treatment only. In females, MP treatment attenuated fluid intake only in very early treatment weeks. Additionally, MP dose-dependently reduced overall fluid intake in males (LD: 7.9%; HD: 14.7%). Differences in fluid intake by the MP-treated rats may be attributable to aversion to taste or the psychophysiological effects of the drug (assuming association between consumption and drug effects were made), or direct effects on thirst; however, the latter is uncorroborated by prior studies that demonstrate no significant changes in fluid consumption in response to MP via intraperitoneal injection (Conners, 1975; Barone et al., 1979; Rajala et al., 2012).

MP treatment reduced food intake in some very early treatment weeks, which is in agreement with prior studies (Goldfield et al., 2007; Gray et al., 2007). During later treatment weeks, however, MP dose-dependently increased food intake in females, and total food consumption was increased in HD MP treated females (19.3%). Despite no differences or an increase in food consumption in MP rats, treatment resulted in an attenuation of body weight in the HD MP group of both sexes (males: 15.3%; females: 12.4%). These results are in agreement with previous studies, which conclude MP treatment elicits a reduction in body weight (Vanina et al., 2002; Faraone et al., 2008; Thanos et al., 2015). Reduced body weight despite increased food intake may be attributable to metabolic effects of stimulants (Ersche et al., 2013) and/or increases in energy expenditure, as MP treated rats exhibited increased locomotor activity as measured by open field and circadian locomotor behavior.

Circadian locomotor activity was assessed over a 24-h period in the home cage during the last week of MP treatment. HD MP treatment resulted in increased dark cycle circadian activity in both males (48%) and females (124%), with a significantly greater and longer-lasting effect seen in females. When locomotor behavior was assessed hourly, HD males were only affected toward the middle to late period of the dark cycle, while HD MP female rats exhibited increased activity throughout most of the dark phase. LD MP treatment also increased dark cycle activity levels in females (45%), which lasted throughout nearly the entirety of the dark phase, albeit to a lesser a degree than HD treatment. Activity levels of LD treated males remained unaffected. These results indicate that the effects of MP are dose-dependent, with higher doses resulting in greater increases in dark cycle activity. Additionally, females are more sensitive to the hyperactivating effects of the drug compared to males, and it appears that HD treatment in males produces similar locomotor responses as the LD in females. MP produced no differences in activity during the light cycle, nor was there a pattern shift of circadian activity; the only effect of MP seen was an increase in amplitude of activity during the dark cycle. While concerns have been presented that psychostimulant treatment may negatively affect sleep patterns in children and treated patients (Schwartz et al., 2004; Sangal et al., 2006; Lee et al., 2012), these results agree with prior studies in rodents and humans that found no effect of chronic MP treatment on circadian patterns nor multiple other sleep parameters (Tirosh et al., 1993; Kent et al., 1995; Thanos et al., 2015).

In agreement with circadian activity results, HD MP treatment increased measures of horizontal activity (distance traveled and velocity) in the open field in both sexes. In males, this was only significant during later weeks of treatment, while females were affected for the entire duration of treatment. LD MP treatment also increased these measures of horizontal activity in females during later weeks of treatment, while males were unaffected. Within MP treated groups, females were significantly more active than males; within the HD, this effect was significant throughout treatment, while in the LD, this effect was significant during later treatment weeks. While male and female water treated groups had similar levels of activity, HD MP treated males (distance: 36%; velocity: 24%) had increased activity to a similar degree as LD treated females (distance: 52%; velocity: 34%), while activity levels of HD treated females were elevated well-beyond that (distance: 210%; velocity: 117%). Additionally, while water treated males and females had consistent levels of locomotor activity throughout treatment, MP treated animals (except for LD treated males) showed increasing locomotor behavior throughout treatment weeks. This finding could be due to increased sensitivity to the locomotor-activating effects of the drug over development, or could represent sensitization to MP, a demonstrated effect of psychostimulant treatment (Kuczenski and Segal, 2001; Yang et al., 2003), that was clearly most robust in HD MP treated females. Similar trends to horizontal open field activity were seen when assessing MP's effects on rearing behavior. HD MP treatment resulted in increased rearing behavior in both males (events: 12%; time: 5%) and females (events: 63%; time: 24%), while LD MP treatment only increased this behavior in females (events: 13%; time: 35%). Rearing has been regarded as a measure of exploratory behavior, and this finding of MP-induced increases in rearing is in agreement with prior studies (Hughes, 1972; Hughes and Greig, 1976). Taken together, these results suggest that females were much more sensitive to the stimulant effects of MP than males, demonstrated by greater horizontal activity and rearing behavior. The results from this study are in agreement with other studies on sex differences in response to MP and other psychostimulants that found that females showed greater levels of activity and exploration in open field than males when given oral MP (van Haaren and Meyer, 1991; Bethancourt et al., 2011). Females have generally been shown to be more sensitive to MP than their male counterparts, a finding that extends to other psychostimulants such as cocaine (Walker et al., 2001; Carrier and Kabbaj, 2012; Chelaru et al., 2012; Van Swearingen et al., 2013).

In the open field test, relative center distance was increased by HD MP in males only (51%); however, as the measure of relative center distance may be sensitive to overall activity levels, time spent in the center of the arena was also assessed. HD MP increased center time similarly in both sexes (males: 25%; females: 19%), while LD treated animals generally showed no differences in center activity compared to water controls. Interestingly, MP does not appear to have the same sexually dimorphic effect on center activity as on other measures of locomotor behavior. Center activity in the open field has been used as a measure of anxiety (Fernández-Teruel et al., 1992), with increased center activity indicating an anxiolytic response. This interpretation is in agreement with previous studies in rats finding that MP treatment decreases anxiety in the open field and other tests, such as the elevated plus maze (Zhu et al., 2010; Thanos et al., 2015), as well as reports of reduced anxiety in clinically-treated ADHD patients (Barrickman et al., 1995; Bouffard et al., 2003).

While HD MP treatment did reduce social interaction in both sexes, this effect was not significant. Previous studies have shown dose-dependent reductions in social play behavior in response to MP administration (Vanderschuren et al., 2008; Robinson and Bucci, 2014). There was however, a significant difference between males and females in our study, with females spending more time engaging in social interaction. A majority of the literature does report sex differences in social interaction; however, most studies found that males are more socially exploratory, and exhibit less social anxiety, than females as a result of biological differences (Olioff and Stewart, 1978; Stack et al., 2010; Carrier and Kabbaj, 2012). One possible explanation for this discrepancy is that male and female testing utilized different types of social stimuli. Females were paired with an age- and weight-matched conspecific, while males were paired with a much younger and smaller juvenile rat in order to avoid aggression (Van Loo et al., 2003; Gromov and Voznesenskaya, 2013).

Results showed that all groups exhibited intact object recognition memory, and that chronic MP treatment had no effect on degree of exploration of the familiar or novel object. Known effects of MP on novel object recognition-based memory are largely mixed. Studies have shown disrupted novel object exploration as a result of altered recognition memory and/or reactivity to novel objects in response to MP treatment (Heyser et al., 2004; Chuhan and Taukulis, 2006). These studies tie with findings that demonstrate that high concentrations of catecholamines in the prefrontal cortex impair working memory (Murphy et al., 1996; Arnsten, 1997). Other studies had shown therapeutic effects of MP to restore novel objection recognition in transgenic mice with the DARPP-32 gene, a dopamine regulated phosphoprotein, knocked out (Heyser et al., 2013). The lack of effect seen on novel object recognition and social interaction may reflect the fact that testing was performed while rats were on MP. Neuroadaptations may have occurred with long-term treatment that results in normal performance while on MP.

Further investigation is necessary to determine the mechanisms driving observed effects and sex differences in physiological and behavioral responses to MP despite dosages being normalized to body weight. Important to note is that rats in this study were single housed out of necessity to accurately monitor and control treatment dosages. Single housing is a “deprived” environment, which could have created stress for the animals and interacted with MP treatment, perhaps in a sex-dependent way, to contribute to effects on physiology and/or behavior. Males and females could also exhibit differences in MP pharmacokinetics, leading to different concentrations of the drug in the blood and brain. A study in adult rats found that following a 5 mg/kg injection of MP, brain concentrations of the drug were consistently higher in females, with a slower rate of drug clearance (Bentley et al., 2015). A clinical study, however, found that in individuals receiving 0.3 mg/kg MP orally, women had lower MP plasma concentrations, yet were more sensitive to the subjective effects of the drug (Patrick et al., 2007). Another possibility is that behavioral differences could be due to previously reported sex differences in catecholamine (particularly dopamine) neurotransmitter, receptor, and transporter levels in the brain (Pohjalainen et al., 1998; Andersen and Teicher, 2000; Lavalaye et al., 2000; Staley et al., 2001). Additionally, clinical studies have found that males and females differ in the amount of dopamine released in response to psychostimulants (Munro et al., 2006; Riccardi et al., 2006). Further studies are necessary to elucidate the biological mechanisms driving the observed sex differences in behavioral responses to chronic oral MP treatment.

## Conclusions

With the increasing diagnoses of ADHD and psychostimulant prescription rates, as well as concern about the illicit use of MP by both adolescents and adults, there is a need to assess the possible physiological and behavioral effects of long-term treatment. Of interest is the investigation of sex differences in the sensitivity to these effects. The results from this study show that chronic MP exposure leads to alterations in body weight, food consumption, locomotor activity, and measures of exploration and anxiety, with no effect seen on social interaction or novel object recognition. Females were particularly more sensitive to the locomotor-activating effects of MP, as well as increases in exploratory behavior. For these measures, a LD of MP in females generally had an equivalent effect of HD treatment in males, despite dosages being normalized for body weight. The HD in females had an even more pronounced effect, while LD treated males generally exhibited no difference in behavior compared to controls. In conclusion, these results provide a critical foundation for further animal studies to examine the effects of chronic MP administration. Future studies should aim to determine the underlying neurobiological mechanisms in MP-induced changes in behavior, particularly in regards to the sex differences observed in the current study. Also of interest is to examine whether behavior is altered following chronic treatment when rats are not presently on the drug (i.e., during an abstinence period), as this is of great clinical relevance for adults who were previously treated with psychostimulants as children.

## Ethics statement

Approved by the IACUC at University at Buffalo.

## Author contributions

Designed study: PT. Wrote the paper and interpreted findings: LR, PT, DK, and MH. Conducted the experiments: LR, MM, JG, DF, EM, CL, AM, MV, JL, and SP.

## Funding

This work was supported by the National Institute of Child Health and Human Development [R01HD70888].

### Conflict of interest statement

The authors declare that the research was conducted in the absence of any commercial or financial relationships that could be construed as a potential conflict of interest.
